# Neural signatures of emotional inference and experience align during social consensus

**DOI:** 10.21203/rs.3.rs-3487248/v1

**Published:** 2023-11-17

**Authors:** Marianne Reddan, Desmond Ong, Tor Wager, Sonny Mattek, Isabella Kahhale, Jamil Zaki

**Affiliations:** Stanford University; University of Texas at Austin; Dartmouth College; University of Oregon; University of Pittsburgh; Stanford University

## Abstract

Humans seamlessly transform dynamic social signals into inferences about the internal states of the people around them. To understand the neural processes that sustain this transformation, we collected fMRI data from participants (N = 100) while they rated the emotional intensity of people (targets) describing significant life events. Targets rated themselves on the same scale to indicate the intended “ground truth” emotional intensity of their videos. Next, we developed two multivariate models of observer brain activity– the first predicted the “ground truth” (*r* = 0.50, *p* < 0.0001) and the second predicted observer inferences (*r* = 0.53, *p* < 0.0001). When individuals make more accurate inferences, there is greater moment-by-moment concordance between these two models, suggesting that an observer’s brain activity contains latent representations of other people’s emotional states. Using naturalistic socioemotional stimuli and machine learning, we developed reliable brain signatures that predict what an observer thinks about a target, what the target thinks about themselves, and the correspondence between them. These signatures can be applied in clinical data to better our understanding of socioemotional dysfunction.

## Introduction

Healthy social functioning increases life expectancy (Holt-Lunstad et al., 2010; Yang et al., 2016), buffers cognitive decline (Cacioppo & Cacioppo, 2014; Fakoya et al., 2020; Landeiro et al., 2017; Read et al., 2020; Ren et al., 2023), improves mental health conditions like depression (Luanaigh & Lawlor, 2008), and enriches a person’s daily life (Goodwin et al., 2001). The quality of a social interaction is shaped by two essential behaviors–how we *signal* our emotions and how we *infer* the emotions of others (Mehu & Scherer, 2012). People who make clear signals and accurate inferences tend to have healthy adolescent adjustment, stable relationships, and high subjective well-being (Gilbert et al., 2020; Hall et al., 2009). Conversely, people who make ambiguous signals and inaccurate inferences are more likely to experience social isolation, or meet criteria for clinical conditions like Autism Spectrum Disorder (ASD; Cassidy et al., 2014), schizophrenia (Green et al., 2008; Takano et al., 2017; Vucurovic et al., 2020), social anxiety (Button et al., 2012; Hirsch & Mathews, 2000), and borderline personality disorder (Baez et al., 2015; Dziobek et al., 2011). Therefore, a developed understanding of how the brain forms socioemotional inferences is clinically advantageous and has the potential to reduce loneliness and improve public health.

The neural processes that lead to conscious inference are computationally complex and require the integration of multiple sources of information such as an observer’s internal homeostatic state, their past experiences, expectations, and social schemas (Chang et al., 2021; Pugh et al., 2022). Neuroimaging studies have implicated the amygdala, medial prefrontal cortex (mPFC), temporoparietal junction (TPJ), and precuneus (Skerry & Saxe, 2014; Spunt & Adolphs, 2019; Zaki et al., 2009) in socioemotional inference; however, integrated models of how multiple brain regions interact with one another to form an inference are lacking. Prior research also fails to disentangle signal *intent* from *inference*. This is partly because the “ground truth” of the signaler’s intent is not available when stimuli are derived from normative data sets or actors, instead of real people. Previous work from our lab compared observer inferences to storytellers’ self-reported “ground truth” ratings and found that accuracy is associated with higher activation in the inferior parietal lobule (IPL), premotor cortex, superior temporal sulcus (STS), and mPFC (Zaki et al., 2009). Accuracy, however, is not a neural process, but an outcome of social signal processing. To better characterize how the brain processes social signals and forms a socioemotional inference, it is necessary to disentangle a target’s “ground truth” intent from an observer’s inference and develop multivariate models of brain activity that can be combined to explain an individual’s inference accuracy.

Naturalistic stimuli are necessary for the study of social signal processing (Zaki & Ochsner, 2009). Though static and unimodal stimuli are useful for isolating specific components of emotion perception and induction, low dimensional stimuli forgo the complexity of the dynamic environments we live in and limit our ability to study social behavior (Eickhoff et al., 2020; Jääskeläinen et al., 2021; Zaki & Ochsner, 2009). Naturalistic audiovisual recordings allow us to capture the subtleties of nonverbal communication, such as nuanced facial expressions, tone of voice, and body language. Moreover, naturalistic stimuli reduce demand characteristics (Grimm, 2010) and allow us to examine the context in which social interactions occur. For instance, studying how people recognize distress in real-life situations provides a better understanding of empathic inference than asking people to rate emotions in a hypothetical situation. First-person narratives, such as storytelling, are particularly opportune stimuli for the study of real-world social interactions. Such stimuli can be sufficiently constrained in the laboratory by limiting spurious background activity while still allowing for the natural unfolding of dynamic socioemotional signaling (Ong et al., 2021). Overall, studying naturalistic stimuli can lead to a more accurate understanding of real-world social interactions, which can inform the development of treatments for social and emotional disorders.

**Figure 1:**
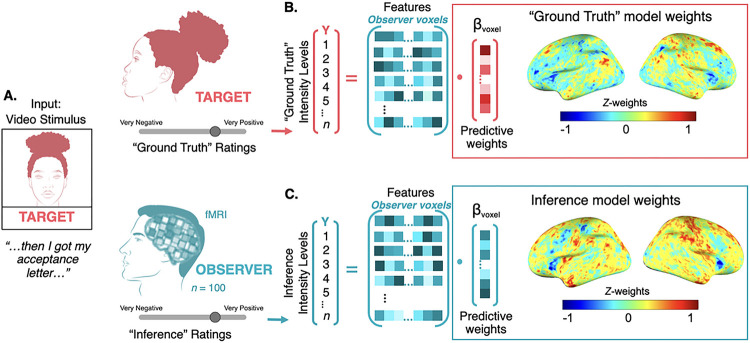
Training the neural models of “ground truth” and inference **A. Paradigm schematic.** Targets recorded themselves telling emotionally significant personal stories, then rated their own videos on a bivalent scale. Target self-ratings served as the “ground truth” of the socioemotional signals conveyed in the videos. Observers viewed these videos in the MRI and rated what they thought the target felt, moment-by-moment, on the same scale. These are the “inference” ratings. Both sets of ratings were subsequently transformed into five (valence independent) levels of intensity. **B**-**C. Model Training**. Two models were trained from the same video stimuli: One aimed to characterize the “ground truth,” and the other aimed to characterize the observer’s inferences. First, brain activity for each intensity level, within each participant, was averaged into a single beta map (these voxels comprised the model’s features). Next, a multivariate LASSO-PCR model was trained to predict both “ground truth” and inference intensity levels (Y = 1 to 5, 5 being the highest intensity) from the corresponding whole brain beta maps (features). Models were trained using leave-one-participant-out cross-validation (LOO-CV). Plotted on the surface maps are the unthresholded predictive *Z*-weights for each model.

To improve our understanding of how an observer’s brain perceives signals and forms inferences in naturalistic contexts, we collected videos of people (targets) describing emotional events in their lives. After being recorded, targets provided moment-by-moment ratings of how they felt as they spoke. These self-reports constitute the “ground truth” of their social signals. We then scanned a second set of participants (observers) with fMRI while they viewed targets and rated what they thought the target was feeling at each moment on the same scale as targets themselves (see Fig. 1). These ratings constitute the observer’s dynamic inferences. Correlations between observer inference and target self-report served as a measure of “empathic accuracy,” or agreement between two people about what one of them feels. We then used machine learning to develop two neural signatures of socioemotional processing– one indicative of the signaler’s *intent* (i.e., the “ground truth”) and one indicative of the observer’s *inference*– and tested if their correspondence is related to empathic accuracy. This work extends our understanding of how the human brain forms socioemotional inferences in three novel ways. First, we use high quality and well-curated naturalistic stimuli that reflect people’s real-world experiences. Second, we use multivariate methods to develop integrated brain models of socioemotional processing. Finally, we investigate the “ground truth” intentions of social targets instead of relying solely on observer inferences to understand an observer’s behavior. This allows us to analyze how a target’s intended signals are perceived, and how that initial perception is related to the observer’s conscious inference. Together, this work advances our scientific understanding of the brain processes that support complex, real-world emotion inference.

## Results

### The “ground truth” intended emotional intensity of another person’s social signals can be predicted from an observer’s brain activity

**Figure 2:**
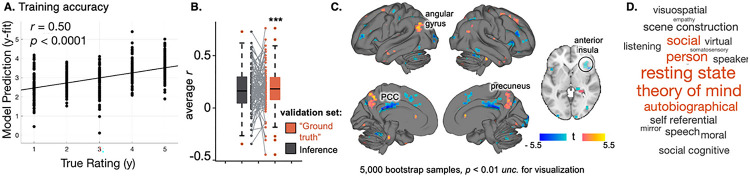
“Ground truth” model of socioemotional intent **A. “Ground truth” model’s cross-validated training accuracy**. A prediction-outcome correlation was calculated across each training fold. The target’s true intensity rating associated with the beta map (y) is plotted on the x-axis. The model’s prediction of the intensity rating associated with the beta map (y-fit) is plotted on the y-axis. The model was able to predict the target’s self-reported, or “ground truth,” emotional intensity from the observer’s brain (*r*= 0.50, *p* < 0.0001). **B. Model validation**. The model was validated by calculating the prediction-outcome correlation across five levels of “ground truth” intensity within participants in held-out validation trials (see Figure S2). Average *r* values for each participant (N = 100) are plotted on the y-axis (orange bar). The model is sensitive to its validation set (*t*(99) = 9.65, *p* < 0.0001). To test its specificity, we repeated this analysis in the inference validation set (gray bar). The “ground truth” model fit the “ground truth” validation set better than the inference set, but the difference was not significant. Data are represented in box plots where the median is a black line and the upper and lower ‘whiskers’ represent the bounds of the quartiles. **C. Thresholded “ground truth” model weights**. The model’s voxel-weight map (Figure 1B) is loosely thresholded (*p* < 0.01 *unc*.) and plotted for visualization (see FDR thresholded regions in Table S1). Activity in right visual and right anterior insular cortices, as well as right angular gyrus, left posterior cingulate cortex (PCC), bilateral precuneus, and bilateral superior frontal gyrus were most important for this prediction. **D. Associated NeuroSynth terms**. The unthresholded predictive weight map was fed to the NeuroSynth topic map decoder. A word cloud was constructed of the top 20 terms (excluding singular brain regions). Word size is scaled by strength of similarity. The terms suggest that this brain pattern is capturing information related to the comprehension of socioemotional schemas.

We developed a novel whole-brain fMRI pattern that can predict, from an observer’s brain activity (N = 100), the “ground truth” intended emotional intensity of the target (Fig. 1B). The model’s average within-subject correlation between predicted and actual ratings was *r* = 0.50 ± 0.02 (standard error), p < 0.0001 (Fig. 2A). It was developed using least absolute shrinkage and selection operator-regularized principal components regression (LASSO-PCR; see STAR methods) with leave-one-subject-out cross validation (LOO-CV). The accuracy of our model is on par with other emotion models in the literature (see Figure S4) and our model’s validity was verified in a held-out validation set (average *r* = 0.19 ± 0.002; *t*(99) = 9.65, *p* < 0.0001; see Fig. 2B). To test its specificity, we repeated this analysis in the inference validation set. The “ground truth” model fit the “ground truth” validation set better, but the difference was not significant (average *r* = 0.18 ± 0.002; *t*(99) = 0.48, *p* = 0.63) in a two-tailed paired *t*-test. Note the stimuli in the validation set are unimodal dynamic stimuli (audio-only and visual-only), while the training data are multimodal (audiovisual). Further testing of this model on audiovisual and other socioemotional stimuli is necessary to determine its out-of-sample sensitivity and specificity.

Our model’s ability to decode the target’s self-reported emotional state indicates that there is a latent representation of a social signal’s “ground truth” in the brains of observers. This latent representation is comprised of a distributed pattern of brain activity that relies most significantly on activity in the right visual and anterior insular cortices as well as the right angular gyrus, left posterior cingulate (PCC), bilateral precuneus, and bilateral superior and inferior frontal gyri (5,000 bootstrap samples; FDR corrected *q* < 0.05; Fig. 2C; see Table S1 for complete list).

We compared the unthresholded brain pattern to other brain maps in the NeuroSynth database (Yarkoni et al., 2011) to characterize functions associated with this network. The “ground truth” pattern is largely unique from previously published beta maps but is most similar to those characterizing brain activity related to ‘resting state,’ ‘theory of mind,’ ‘person,’ ‘social, ‘autobiographical,’ beliefs,’ ‘spatial,’ ‘[scene] construction,’ ‘speech,’ and ‘self-referential processing,’ respectively (Fig. 2D). The unthresholded brain pattern was also compared to well-validated brain-based models of emotion, empathy, and interoception to further gauge its sensitivity and specificity the “ground truth” intensity of a target’s social signal (see STAR Methods and Figure S5). The “ground truth” model was dissimilar from emotion models that predict how an observer feels in response to an image or event (PINES *cosine similarity* = −0.03; Chang et al., 2015; NPS *cosine similarity* = −0.03; Wager et al., 2013; social rejection *cosine similarity* = −0.04; Woo et al, 2014). However, it was weakly similar to naturalistic models of empathic care (*cosine similarity* = 0.06) and distress (*cosine similarity* = 0.10; Ashar et al., 2016). Overall, this suggests that the “ground truth” brain pattern is not reducible to the general processing of affect but is capturing information related to the comprehension of dynamic socioemotional schemas.

### An observer’s socioemotional inference can be predicted from their brain activity

**Figure 3:**
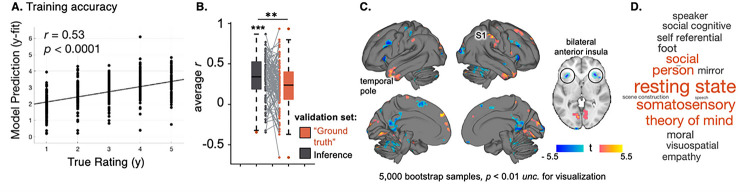
Inference Model **A. Inference model’s cross-validated training accuracy.** The observer’s intensity rating associated with the beta map (y) is plotted on the x-axis. The model’s prediction of the intensity rating associated with the beta map (y-fit) is plotted on the y-axis. The model was able to predict an observer’s inference from their brain activity (*r* = 0.53, *p* < 0.0001). **B. Model validation**. The inference model was verified in its held-out validation trials (*t*(99) = 12.48, *p* < 0.0001). Average *r* values for each participant (N = 100) are plotted on the y-axis (gray bar). To test its specificity, we repeated this test in the “ground truth” validation set (orange bar). The inference model fits its own validation set better than the “ground truth” validation set (*t*(99) = 2.77, *p* = 0.007). Data are represented in box plots where the median is a black line and the upper and lower ‘whiskers’ represent the bounds of the quartiles. **C. Thresholded inference model weights.** The voxel-weight map is liberally thresholded (*p* < 0.01 *unc.*; see Figure 1C) and plotted for visualization (see Table S2 for FDR thresholded regions). Thresholding revealed activity in the cerebellum (bilateral crus), precuneus, primary somatosensory cortex (S1), inferior frontal gyrus, bilateral superior medial frontal gyrus, lingual gyrus, temporal pole, and anterior insular cortex were most important for this prediction. **D. Associated NeuroSynth terms.** The terms in the Neurosynth database most similar to the inference model are plotted as a word cloud and scaled by strength of similarity. The terms suggest this brain pattern is related to mentalizing.

We identified a pattern of brain activity that correlated with the level of intensity observers (N = 100) ascribed to storytellers on a moment-to-moment basis (Fig. 1C). The model’s average within-subject correlation between predicted and actual ratings was *r* = 0.53 ± 0.02, *p* < 0.0001 (Fig. 3A). This model was trained using LOO-CV LASSO-PCR to predict observer’s *inferences* (see STAR Methods). The inference model was verified in its held-out validation set (average *r* = 0. 32 ± 0.002; *t*(99) = 12.48, *p* < 0.0001). The model was specific to observer inferences– it’s performance on its own validation set (*r* = 0.23 ± 0.002) was significantly higher than its performance on the “ground truth” validation set in a two-tailed paired t-test (*t*(99) = 2.77, *p* = 0.007, CI = [0.03–0.16], Cohen’s *d* = 0.33; Fig. 3B).

The brain regions that most significantly contributed to the formation of socioemotional inferences include the cerebellum (bilateral crus), left precuneus, right primary somatosensory cortex, right inferior frontal gyrus, bilateral superior medial frontal gyrus, bilateral lingual gyrus, bilateral temporal pole, and bilateral anterior insular cortex (5,000 bootstrap samples; FDR corrected *q* < 0.05; see Table S2 for complete list; see Fig. 3C thresholded at *p* < 0.01 *uncorrected*).

Again, we compared the unthresholded brain pattern to other published brain maps to characterize functions associated with this network. The networks and functions in the NeuroSynth database which were most similar to the inference model were: ‘resting state,’ ‘somatosensory,’ ‘person,’ ‘theory of mind,’ ‘social,’ ‘spatial,’ ‘foot,’ ‘moral’, ‘self-referential,’ and ‘beliefs,’ respectively (Fig. 3D). The inference model was unique from the “ground truth” model in that it was associated with maps related to somatosensory simulation and bodily action. When we compared the inference model to the brain-based models of emotion, empathy, and interoception in Figure S5, we found that like the “ground truth” model, the inference model is unique from existing signatures but has a small positive cosine similarity with models of dynamic human-to-human empathic processing. Overall, these comparisons indicate that our model of socioemotional inference draws largely from mentalizing networks.

### Neural patterns underlying “ground truth” signal intent and inference are dissociable

Both the “ground truth” and inference models were developed on observer brain activity, however, they reveal dissociable components of socioemotional processing (*cosine similarity* = 0.29). Cosine similarity ranges from − 1 to 1, where values closer to 0 indicate the two vectors are orthogonal to each other. When the value is closer to 1, images are more similar. When the value is closer to −1, images are more opposite. To further investigate their dissociability, we assessed general overlap of voxels that significantly contribute to each model’s performance. After thresholding, the only voxel-overlap was in the right anterior insula (Figure S6), however, voxel-wise patterns in the bilateral insular cortex (mask from Harvard-Oxford Atlas) were unique (c*osine similarity* = 0.22). Together, these metrics indicate that the two models capture unique components of socioemotional brain activity.

**Figure 4:**
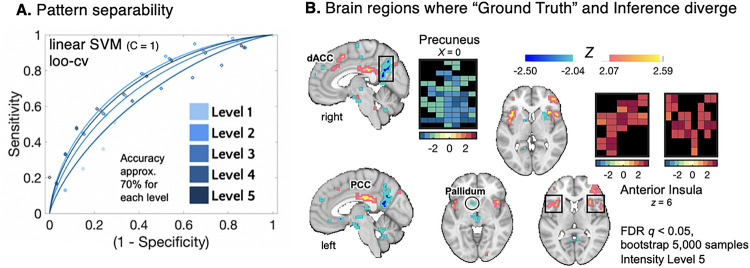
Separability of “ground truth” and inference activity patterns in the brain **A. Model separability** We tested if bran activity patterns related to the “ground truth” of the socioemotional information and the observer’s inference are separable at each level of stimulus intensity. Binary SVMs revealed pattern separability at each level. Receiver operating curves (ROC) visualize the sensitivity and specificity of the SVMs which separated the patterns. **B. Brain regions where “ground truth” and inference diverge**. To better understand the brain regions that distinguish between “ground truth” and inference, the predictive voxel weights for the SVM classification at Level 5 (high intensity) were bootstrapped (5,000 samples) and thresholded (FDR *q* < 0.05). Patterns of activity in the dorsal anterior cingulate (dACC), posterior cingulate (PCC), pallidum, insula, and precuneus maximally separate “ground truth” and inference at the highest level of socioemotional intensity.

Next, we tested if bran activity patterns related to “ground truth” and inference are separable at each level of stimulus intensity. To do this, we trained a binary linear SVM (see STAR Methods) to separate subject-level “ground truth” and inference maps at each level of rating intensity. Each classification accuracy was significantly greater than chance (*level 1*: Acc. = 70.00% ±3.3%, p < 0.0001, Area Under the Curve (AUC) = 0.69; *level 2*: Acc. = 69.00% ±3.3%, *p* < 0.0001, AUC = 0.75; *level 3*: Acc. = 70.00% ±3.3%, *p* < 0.0001, AUC = 0.73; *level 4*: Acc. = 69.00% ± 3.3%, *p* < 0.0001, AUC = 0.68; *level 5*: Acc. = 70.00% ±3.3%, *p* < 0.0001, AUC = 0.75) indicating that the “ground truth” and inference activity patterns are linearly separable, and therefore, represent unique neural processes (Fig. 4A). To better understand the brain regions that distinguish between “ground truth” and inference, the predictive voxel weights for the classifier trained at the highest level of intensity (level 5) was bootstrapped (5,000 samples) and thresholded (FDR *q* < 0.05) so that the brain regions where “ground truth” and inference maximally diverge could be compared (Fig. 4B). Patterns of activity in the dorsal anterior cingulate (dACC), PCC, anterior insula, pallidum, and precuneus maximally separate “ground truth” and inference at the highest level of socioemotional intensity. These regions correspond with those that diverge at the lowest level of intensity (see Figure S7). Together, these analyses indicate that the multivariate patterns which comprise the “ground truth” and inference models are dissociable from one another and therefore reflect unique, but simultaneous, components of socioemotional processing.

### When the brain-based models of a social signal’s “ground truth” and an observer’s inference align, observers are more empathically accurate

**Figure 5:**
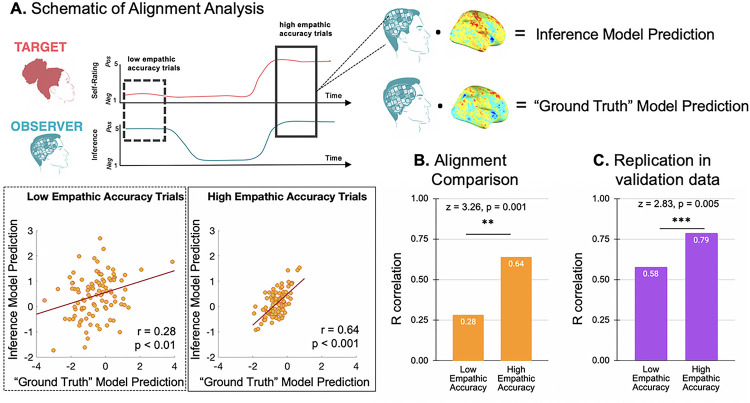
Increased alignment between the “ground truth” and inference models is related to empathic accuracy **A. Schematic of alignment analysis**. To test how each model’s predictions relate to an observer’s empathic accuracy, we took the dot-product between each model and individual subject’s (N = 100) maps of empathic accuracy at two levels: low and high accuracy, and then added in the models’ intercepts. **B. Alignment comparison**. To test how the alignment of the “ground truth” and inference patterns are related to empathic accuracy, we correlated each model’s predictions across all participants. When participants are inaccurate, there is more variance across the predictions of the “ground truth” and inference models, and therefore they are weakly correlated (*r* = 0.28, *p* < 0.01). However, when participants are highly accurate, there is lower variance between the model predictions, and they are better correlated (*r* = 0.64, *p* < 0.001). The alignment between the two models was significantly greater during high accuracy performance than low accuracy performance (z = −2.71, *p*= 0.003). **C. Replication in validation data**. We verified this effect in the validation trials (two-tailed z-test of the correlation difference *z* = 2.83, *p* = 0.005). This analysis indicates that our models of brain activity underlying the “ground truth” of the socioemotional signal and the observer’s inference can be combined to predict an observer’s empathic accuracy.

After we identified these two unique components that underlie socioemotional processing, we sought to test how they interact in relation to an individual person’s empathic accuracy. To do this, we applied the “ground truth” and inference models to participant-level brain activity when participants made inaccurate inferences (low empathic accuracy) and accurate inferences (high empathic accuracy; see Fig. 5A and STAR Methods for details). Then we correlated the predictions of the two models across all participants. When participants are inaccurate, there is more variance across the predictions of the “ground truth” and inference models, and, therefore, they are weakly correlated (*r* = 0.28, *p* < 0.01; Fig. 5A). However, when participants are highly accurate, there is less variance between the model predictions, and they are highly correlated (*r* = 0.64, *p* < 0.001). The alignment between the two models was significantly greater during high accuracy performance than low accuracy performance (*z* = −2.71, *p* = 0.003; Fig. 5B). We verified this effect in the validation trials (low empathic accuracy alignment *r* = 0.58, *p* < 0.0001; high empathic accuracy alignment *r* = 0.79, *p* < 0.0001; two-tailed z-test of the correlation difference *z* = 2.83, *p* = 0.005; Fig. 5C). This analysis indicates that when an observer is making accurate inferences, both the neural patterns underlying the “ground truth” of the socioemotional signal and the observer’s inference are highly similar to the observer’s brain activity at that moment. That is, greater concordance between the latent representation of the target’s intended signal intensity and the observer’s inference indicates greater empathic accuracy.

### Brain regions associated with the divergence from the “ground truth” during inaccurate inference

**Figure 6:**
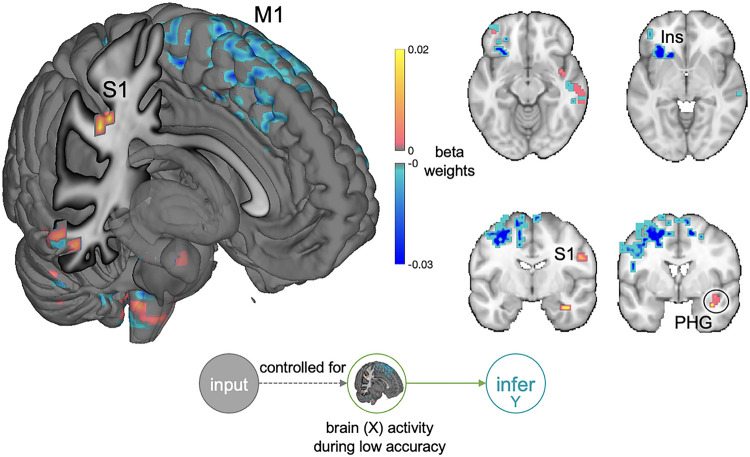
Exploratory analysis of brain regions uniquely related to the inference model’s pattern expression during low accuracy trials In this exploratory univariate analysis, brain activity during low accuracy trials (see schematic in Figure 5) was used to predict the pattern expression of the inference model while controlling for the pattern expression of the “ground truth” model. Plotted are the significant beta weights (FDR *q* < 0.05, k = 25). When a person makes an incorrect inference that diverges from the “ground truth” model’s prediction, activity in the right primary somatosensory cortex (S1) and parahippocampal gyrus (PHG) increases, while activity in the left insula (Ins) and primary motor cortex (M1) decreases. The unthresholded map of activity is dissimilar from maps of finger tapping in the NeuroSynth database (*cosine similarity* = −0.07). One possible interpretation of these exploratory results is that somatosensory simulations drive the transformation of latent “ground truth” information into a conscious inference.

In a post-hoc exploratory univariate analysis, brain activity during low accuracy trials (see schematic in Fig. 5A) was used to predict the pattern expression of the inference model while controlling for the pattern expression of the “ground truth” model. ‘Pattern expression’ is the dot-product between two multivariate patterns (see STAR Methods for details). Low accuracy trials were selected so we could isolate regions where inference-related activity maximally diverged from latent “ground truth”-related activity. The resulting model weights were corrected for multiple comparisons (FDR *q* < 0.05, *k* = 25). Positive clusters were revealed in the right primary somatosensory cortex (S1) and right parahippocampal gyrus (Fig. 6). Negative clusters were revealed in the left insula and left primary motor cortex (M1). This activity is dissociable from maps of finger tapping in the NeuroSynth database (*cosine similarity* = −0.07). These results suggest that activity in S1, M1, and parahippocampal gyrus contribute to the formation of a dynamic socioemotional inference, above and beyond the latent “ground truth” representation; albeit, here, activity in these regions contribute to the formation of an incorrect inference.

## Discussion

The effectiveness of social signaling depends upon both the intended content of the signal (i.e., the socioemotional “*ground truth*”) and the interpretation of the signal by an observer (i.e., socioemotional *inference*; Mehu & Scherer, 2012). To better understand how social signaling is processed in the brain, we sought to dissociate the neural patterns underlying “ground truth” and inference in a dynamic naturalistic storytelling paradigm and test how these patterns relate to empathic accuracy. We found that both “ground truth” (i.e., the target’s self-reported emotional intensity) and an observer’s inferences about the target’s emotional intensity could be predicted from observers’ brain activity. The multivariate brain patterns derived from these predictions are dissociable; however, when the models’ predictions align, observers make more accurate inferences. These findings suggest that there is some latent representation of a target’s “ground truth” emotional intensity that observers transform into conscious inference. Moreover, these findings suggest that participants quickly and accurately represent another’s mental state even when they make an incorrect inference.

How can it be possible that participants “represent” the “ground truth” of another person’s social signals and that this “representation” can be dissociable from the participants’ conscious inferences? Human adults have highly adept schemas of social information that are activated when they perceive prototypical socioemotional expressions (Izard, 2007). Prior work has found that rich, category-specific visual features can be readily mapped to distinct emotions that are coded across many brain regions including primary visual cortex (Kragel et al., 2019). Unless a target is intentionally trying to deceive an observer, or has a disorder that impacts socioemotional communication, a target will convey information in a manner that will activate the correct schema in an observer (Chang et al., 2021). That is the function of social signaling– to convey information in a manner that will be quickly and accurately understood by the individual an organism wishes to convey it to (Mehu & Scherer, 2012). We suspect that the “ground truth” pattern revealed in this investigation is capturing this process of schema-activation in the observers. Schema-activation in this investigation is (a) specific to the *intensity* of a signal and (b) highly dynamic and multimodal.

The “ground truth” pattern relies significantly on brain areas implicated in speech comprehension (right angular gyrus; Seghier, 2013) and scene construction (PCC and calcarine sulcus; Irish et al., 2015), as well as mentalizing and social cognition (bilateral superior frontal gyrus, precuneus, and anterior insula; Van Overwalle & Baetens, 2009). It does not, however, rely on the amygdala– a brain region heavily implicated in emotion perception (Spunt & Adolphs, 2019). The whole brain “ground truth” pattern is also unique from PINES, which is a multivariate pattern that can predict the emotional intensity of negative static images with high accuracy (Chang et al., 2015). PINES was trained on emotional images, only some of which were social. Therefore, it is possible that our “ground truth” pattern is specific to the processing of *socioemotional* schemas. This pattern may be something the brain constructs in dynamic social interactions that helps it to create a stable representation of the social target. This interpretation is inspired by constructionist theories of emotion, which state that emotions are not fixed entities that have distinct and specific brain circuits, but flexible processes that are inseparable from the context in which they emerge (Barrett, 2017; Russell, 2003).

It is important to note that none of the subcortical regions commonly implicated in emotion perception (i.e., the amygdala, striatum, and periaqueductal gray) were significantly weighted in the “ground truth” or inference models. Instead, the distributed patterns primarily included cortical regions that perform complex multisensory integration (i.e., angular gyrus, frontal gyrus, PCC, temporal pole, and the precuneus; Scheliga et al., 2022). From a constructionist viewpoint, the socioemotional “ground truth” pattern may reflect neural processing that abstracts the literal input (i.e., facial expressions, body movements, speech and vocal intonations) into socioemotional schemas related to intensity. Similarly, the inference model may represent a subsequent stage of processing, where the information the target signaled is related to the observer’s past and current experiences and their expectations for the future. Indeed, the inference pattern relied both on mentalizing networks and brain areas implicated in social abstraction and somatosensory processing– the temporal pole and S1, respectively (Quandt et al., 2017). Being that the “ground truth” and inference models were (1) dissociable, (2) verifiable in their own held-out validation sets, and (3) unique from other published models of emotion induction, like PINES, we suspect that they capture unique components of social signal processing– schema activation and deliberate inference formation– that can only be experimentally-evoked by dynamic, naturalistic stimuli.

When the “ground truth” and inference models’ predictions aligned in the brains of the observers, observers made more accurate inferences. Furthermore, the two unique models could be combined to predict the empathic accuracy of observers in held-out validation trials. Interestingly, when individuals made inaccurate inferences, activity in right S1 and PHG increased with the intensity of their inferences when controlling for the intensity of the “ground truth” representation. This suggests that somatosensory simulations may support the transformation of socioemotional “ground truth” into a conscious, reportable inference. Part of the transformation from “ground truth” to inference in this paradigm requires a motor response: Observer’s must update their ratings with a button press. Therefore, it is difficult to disentangle this brain pattern from button pressing entirely; however, this pattern of brain activity was dissimilar from patterns associated with finger tapping in the NeuroSynth database. Furthermore, though the left lateralization of activation in M1 was consistent with the (right) hand making the ratings, the positive clusters in S1 and the PHG were ipsilateral to the ratings-hand, suggesting this activity is not reducible to task-induced motion. This is consistent with prior research that shows S1 activates during motor imagery and empathy (Blakemore et al., 2005; Hooker et al., 2010; Jafari et al., 2020; Schaefer et al., 2020). Furthermore, prior studies of imagination have found right lateralization of imagined stimuli (Reddan et al., 2018). Indeed, a growing thrust of empathy research purports a role for somatosensory simulations in the understanding of social interactions (Genzer et al., 2022; Jospe et al., 2020, 2022). As for the PHG, activity in this region is implicated in episodic memory and the processing of scenes (for a review see Aminoff et al., 2013). Taken together, these results suggest that inference involves an internalizing of the events described by the target, and that people simulate the actions described and relate them to their own prior experiences and expectations.

This study has several limitations. First, the “ground truth” and inference ratings occur spontaneously and sometimes simultaneously in this paradigm, therefore, we are unable to directly model the transformation of “ground truth” into inference in the brain. Second, though the stimuli themselves are highly dynamic and complex, the models are trained to predict only a single dimension of socioemotional information: intensity. This was done because naturalistic stories often signal positive and negative information at a faster rate than we can sample the brain data (see Polimeni & Lewis, 2021). For example, a participant may be describing both the sadness and the love they felt after the death of a family member. These are intense, complex emotions, therefore, removing valence from individual ratings allowed us to better model dynamic shifts in emotion signaling and to isolate signatures of signal *intent* from observer *inference*. Further validation of our models on other naturalistic and social audiovisual data is necessary to determine their sensitivity and specificity to both a target’s self-reported internal emotional state and an observer’s conscious inference of that state.

Various neuroimaging studies have attempted to predict aspects of socioemotional processing from human brain activity (see Figure S4 for a summary), however, this is one of the first investigations to situate socioemotional processing within the ethological framework of social signaling and inference. Most existing models were developed to predict an *observer’s* internal emotional state after it was experimentally influenced by an image or story, and the stimuli are often exaggerated social signals (e.g., an image of an angry actor pointing a gun). This is the first investigation to delineate brain activity related to the “ground truth” of social signals from brain activity related to the inferences observers make when they perceive these signals. The dissociability of these processes provides a new foundation for emotion research and delineates new promising targets for clinical intervention.

Sharing information is essential to the well-being of individuals and the communities they are a part of because individuals must interact with each other to achieve personal needs that cannot be achieved alone. Effective signaling can engender social bonds, mutual aid, and collaboration (Mauss et al., 2011). Ineffective signaling, however, can be costly. A missed alarm call can result in death, while misunderstanding social signals can result in ostracization or rejection. People with ASD experience this from both ends: They have difficulty understanding the intentions, thoughts, and feelings of others *and* have difficulty being understood. As a result, people with ASD experience high levels of loneliness and social isolation relative to other disability groups (Causton-Theoharis et al., 2009; Mazurek, 2014). However, little research emphasis has been placed on people’s ability to decode the social signals of people with ASD. The current study creates a new avenue for such investigations because it allows researchers, for the first time, to disentangle a signal’s intent from an observer’s inference, in dynamic real-world situations.

In summary, our study delves into the intricate processes of how humans interpret dynamic social signals and make inferences about the internal states of others. Using naturalistic stimuli, fMRI data, and machine learning, we established two distinct neural signatures: one predicting the “ground truth” intended emotional intensity of a target and another predicting an observer’s inferences. These neural patterns are dissociable, indicating that they are separate components of socioemotional processing. Notably, when these brain-based models align, individuals demonstrate higher empathic accuracy. This work offers valuable insights into the brain processes that underlie the interpretation of social signals and has the potential to inform treatments for social and emotional disorders.

## Methods

### CONTACT FOR RESOURCE SHARING

Further information and requests for resources and code should be directed to and will be fulfilled by the Lead Contact, Marianne Reddan (marianne.reddan@einsteinmed.edu).

### EXPERIMENTAL MODEL AND SUBJECT DETAILS

One hundred (59 Women, 37 Men, 4 No Response, average age = 25.23 STD = 9.96) adult healthy members of the Stanford University community participated in this study. Forty-two participants identified as White, 32 as Asian American, 14 as Hispanic/Latinx, 7 as Black, and 5 did not report their race (see Supplementary Fig. 1A). Participants were asked to provide their gender in binary terms (male or female). Participants were asked to complete the MacArthur Scale of Subjective Social Status (Adler et al., 2000). Average perceived socioeconomic status (SES) on the 10-point ladder scale was 6.71 (STD = 1.57). SES was skewed towards the upper and upper middle classes (Mode = 7; Median = 7; Supplementary Fig. 1B). Eighty-nine participants reported having at least some college education (this includes those currently enrolled as Stanford undergraduates). Eighty-three reported their parents had at least some college education. Participants were all right-handed.

Participants were recruited through internal and surrounding communities near Stanford University in Stanford, CA. All participants gave informed consent and were paid for their participation. This study was approved by the Stanford University Institutional Review Board.

### METHOD DETAILS

#### Experimental procedures

##### Eligibility Criteria.

Participants were required to be between the ages of 18 to 65. Participants who had contraindications for the MRI environment were excluded from this study. Sample size was determined via previously published studies of empathic accuracy and narrative storytelling (Zaki et al., 2009).

##### Stimuli.

We selected a subset of 24 videos (19 unique targets or storytellers) from a curated high-quality video dataset of 193 video clips of 49 volunteers describing emotional life events known as the Stanford Emotional Narratives Dataset (SENDv1; Ong et al., 2021). The targets used in this selection were balanced for gender (11 Women and 8 Men) and racial representation (9 White, 5 Black, 4 Asian, and 1 Latinx/Hispanic; see Supplementary Fig. 1A). Many of the targets were current students. “Ground truth” self-ratings of emotional valence were obtained from targets on a moment-by-moment bipolar scale (very negative to very positive). Twelve of the 24 videos featured negative life events, while the other 12 featured positive life events. Videos were cut down in length so that they ranged from 1–3 minutes. Five targets contributed two separate videos (one positive, one negative) to this dataset, however, trials with duplicate targets were not used in the main analysis (see Experimental Session for more information). Stimuli were displayed using Psychtoolbox v3 via MATLAB R2017B.

##### Randomization.

This is a within-subjects design, where stimulus presentation was pseudorandomized in three a priori orders. Each subject was randomly assigned to an order when they signed up for the experiment. Video presentation and intertrial interval (ITI) lengths were shuffled and fixed to an order number.

##### Practice Session.

All participants completed a “practice session” before the experiment where they learned how to make their ratings on the slider provided. Participants viewed 10-s clips of stimuli from the SENDv1 dataset that are unique from those used in the study.

##### Experimental Session.

All 100 participants were shown short movies of first-person emotional narratives while inside the fMRI environment. While viewing, participants rated the emotional-narrative stimuli moment-by-moment on a bivalent scale by way of button presses that moved a slider on screen. Participants pressed the buttons to move it right or left with their index and middle finger, respectively, of their right hand. There were three sensory conditions in this experiment: a visual-only condition, where participants could see but not hear the videos (8 trials); an auditory-only condition where they could hear but not see the videos (8 trials); and an audiovisual condition where they could both see and hear the videos (8 trials). There were 24 trials total, divided into two runs of 12 trials each (See Supplementary Fig. 2A). The audiovisual condition (8 trials) comprises the model training data and is the modality of interest in the present study. The other two conditions were combined and used as a held-out validation set. Due to a coding error, only 23 videos were spread across the audiovisual condition during counterbalancing (the missing video was negatively valenced; see Supplementary Fig. 3 for a depiction of the valence of all the audiovisual stimuli). Five targets contributed two separate videos (one positive, one negative) to this dataset, however, the sensory channel of presentation was not repeated within targets. Videos were separated by an ITI (fixation cross) that ranged from 4–12 s.

##### Post-Experiment Session.

Immediately after the fMRI experiment, participants were shown 10 s clips of each video they watched in the scanner, in the same sensory modality (auditory-only, visual-only, and audiovisual) and order that was presented to them in the scanner. First, participants answered True-False questions about the story to assess comprehension and attention. Next, participants were asked if the target was familiar to them. The targets were recruited on Stanford campus; therefore, it was important to confirm that our participants were not friends or acquaintances with the targets. Participants were also asked to rate the overall emotional valence of the stimulus on the same bipolar scale they used during the experiment. This session was displayed via MATLAB R2017B on an Apple laptop computer in an experiment room in the lab.

### DATA COLLECTION

#### Ratings acquisition

Participants were asked to “rate how positive or negative you think the target is feeling moment to moment” on a bipolar visual analog scale (VAS) scale with anchor points ‘very negative’ to ‘very positive.’ Participants did not see any number responses, but their ratings were recorded on a scale from 0 to 100. Ratings were collected via a 2×4 bimanual button box through the fORP 932 modular response box system. Ratings were collected through Psychtoolbox v3 (http://psychtoolbox.org/) in MATLAB R2017B, and were sampled by Psychtoolbox every 0.5 s. One button press moved the slider by 12 pts. The slider could be moved to the right or left via buttons pressed by the index and middle finger on the participant’s right hand.

#### Neuroimaging acquisition

The study was conducted at the Stanford University Center for Cognitive and Neurobiological Imaging (CNI) using a 3T GE Discovery MR750 scanner (running ESE version 23.1 v02: the GE operating system) and a 32 channel Nova Medical head coil. The scan began with a 3-plane localizer or ‘scout’ scan to find the participant’s head. Next, we collected a 3D T1 weighted anatomical scan at 0.9 mm isotropic resolution (Flip Angle = 12; FOV = 23). This scan uses GE’s “BRAVO” sequence. This was followed by a higher-order shim and a field map was collected before each functional scan. Functional scans used a gradient echo sequence (Full brain BOLD EPI, TR = 2 s, TE = 25 ms, FOV = 23.2, Flip Angle = 77, 46 slices at a 2.9 mm slice thickness, Acquisition Order = Interleaved, voxel size = 2.9mm^3). A 3x in-plane acceleration was used to reduce EPI distortion. Functional image acquisition was divided into two runs. Between runs there was a break of approximately 15–30 s where the experimenter checked the participant to make sure they were comfortable and alert.

### QUANTIFICATION AND STATISTICAL ANALYSIS

#### Outlier removal

No outlier subjects were found nor removed from this dataset. One participant experienced a technical error where they were shown duplicate trials. Duplicate trials were removed from this participant’s data prior to analysis.

#### Preprocessing of the “ground truth” and inference ratings

“Ground truth” and inference ratings were down sampled from (0.5 s) to the rate of the TR (2 s). Next, both sets of ratings were range-normalized within participants (for observers, range normalization was done across all video stimuli). Range-normalization involved subtracting the minimum of that participant’s ratings then dividing by the [max - min] of that person’s ratings, from a rating at any given time point. This is also known as feature scaling, and it yields values that are shifted and rescaled so that all values range between 0 and 1. Next, those ratings were adjusted into 5 levels of valence-independent emotional intensity. That is, ratings between 41–50 and 51–60 were set to level 1; ratings between 31–40 and 61–70 to level 2; ratings between 21–30 and 71–80 to level 3; ratings between 11–20 and 81–90 to level 4; and ratings between 0–10 and 91–100 to level 5. Level 5 is the highest intensity rating. This was done because naturalistic stories often combine positive and negative emotions and can signal positive and negative information at a faster rate than we can sample the brain data. For example, a participant may be describing both the sadness they felt and the love they felt after the death of a family member. These are both intense, complex emotions, so removing valence from individual ratings allows us to better model dynamic shifts emotion signaling and to isolate signatures of signal *intent* from an observer’s *inference*.

#### Derivation of empathic accuracy ratings

Empathic accuracy ratings were constructed by first subtracting the normalized “ground truth” ratings from the normalized observer inference ratings, across the whole time series. These ratings were then converted into 5 levels where the highest level 5 reflects times where there was no difference between the observer’s ratings and the “ground truth;” that is, when observers were most accurate. The lowest level 1 reflects when there was a maximal difference between the observer’s ratings and the “ground truth” in either direction; that is, when observers were least accurate. This measure is not valence independent.

#### Imaging preprocessing

Imaging data were first converted into BIDS via in-house scripts. All data were then preprocessed using FMRIPREP version stable (Esteban et al., 2019; Esteban, Oscar et al., 2023; RRID:SCR_016216), a Nipype (Gorgolewski et al., 2011; Gorgolewski, et al., 2017; RRID:SCR_002502) based tool. Each T1w (T1-weighted) volume was corrected for INU (intensity non-uniformity) using N4BiasFieldCorrection v2.1.0 (Tustison et al., 2010) and skull-stripped using antsBrainExtraction.sh v2.1.0 (using the OASIS template). Brain surfaces were reconstructed using recon-all from FreeSurfer v6.0.1 (Dale et al., 1999; RRID:SCR_001847), and the brain mask estimated previously was refined with a custom variation of the method to reconcile ANTs-derived and FreeSurfer-derived segmentations of the cortical gray-matter of Mindboggle (Klein et al., 2017; RRID:SCR_002438). Spatial normalization to the ICBM 152 Nonlinear Asymmetrical template version 2009c (Fonov et al., 2009; RRID:SCR_008796) was performed through nonlinear registration with the antsRegistration tool of ANTs v2.1.0 (Avants et al., 2008; RRID:SCR_004757), using brain-extracted versions of both T1w volume and template. Brain tissue segmentation of cerebrospinal fluid (CSF), white-matter (WM) and gray-matter (GM) was performed on the brain-extracted T1w using fast (Zhang et al., 2001; FSL v5.0.9, RRID:SCR_002823).

Functional data was slice time corrected using 3dTshift from AFNI v16.2.07 (Cox, 1996; RRID:SCR_005927) and motion corrected using mcflirt (FSL v5.0.9; Jenkinson, 2003). This was followed by co-registration to the corresponding T1w using boundary-based registration (Greve & Fischl, 2009) with six degrees of freedom, using bbregister (FreeSurfer v6.0.1). Motion correcting transformations, BOLD-to-T1w transformation and T1w-to-template (MNI) warp were concatenated and applied in a single step using antsApplyTransforms (ANTs v2.1.0) using Lanczos interpolation.

Physiological noise regressors were extracted applying CompCor (Behzadi et al., 2007). Principal components were estimated for the two CompCor variants: temporal (tCompCor) and anatomical (aCompCor). A mask to exclude signal with cortical origin was obtained by eroding the brain mask, ensuring it only contained subcortical structures. Six tCompCor components were then calculated including only the top 5% variable voxels within that subcortical mask. For aCompCor, six components were calculated within the intersection of the subcortical mask and the union of CSF and WM masks calculated in T1w space, after their projection to the native space of each functional run. Framewise displacement (Power et al., 2014) was calculated for each functional run using the implementation of Nipype.

Many internal operations of FMRIPREP use Nilearn (Abraham et al., 2014; RRID:SCR_001362], principally within the BOLD-processing workflow. For more details of the pipeline see https://fmriprep.readthedocs.io/en/stable/workflows.html.

#### Univariate analysis

Preprocessed BOLD runs were concatenated for each participant. Next a participant-specific design matrix was created. It included 36 nuisance regressors extracted via FMRIPREP. These nuisance regressors included CSF and white matter regressors and their derivatives as well as 3D motion regressors and their derivatives and spikes or motion outliers. Next, the regressors of interest were added to the design matrix and three unique “single trial” models were fit to the observers’ brains.

#### Single Trial Observer Rating Models

Regressors were constructed for each rating level for each video stimulus and input into the participant’s design matrix (see Supplementary Fig. 2B). Because participant’s made individual ratings for each video, the number of regressors for each participant varied. Ratings regressors were convolved using the canonical HRF function from the SPM12 toolbox (https://www.fil.ion.ucl.ac.uk/spm/). The nuisance regressors and intercept were added to each design matrix and then an ordinary least squares (OLS) regression was run for each participant. This entire procedure was repeated three times, for the three unique rating types (“ground truth” target self-ratings, observer interference ratings, and empathic accuracy). Variance Inflation Factors (VIF) were calculated for all regressors of interest to perform data quality control. Event trials with a VIF greater than three standard deviations from an individual’s mean were removed as outliers. We tested for intercorrelation across the design matrix and found no issues of multicollinearity within regressors of interest.

#### Predictive analysis

##### Model Training

Brain activity for each intensity level within each participant was averaged into a single beta map and then a LASSO-PCR model (lasso number = 120; error type = Mean Squared Error (MSE)) was trained, via LOO-CV, to predict the intensity level (1–5). LASSO-PCR is a machine-learning–based regression technique. Due to its penalization method, LASSO regression simultaneously performs feature selection and model estimation (Tibshirani, 1996). We used the standard lasso hyperparameters built into the CANLab toolbox (https://github.com/canlab) for the development of the neurologic pain signature (NPS; Wager et al., 2013) and the picture-induced negative emotion signature (PINES; Chang et al., 2015), and did not tune these parameters during training. This procedure was performed twice. First, for the “ground truth” ratings and second for the observer inference ratings. The results were two brain-based models of “ground truth” signal *intent* and socioemotional *inference*, respectively. Our metric for assessing model accuracy was prediction-outcome correlation (OLS partial r). We used permutation tests to obtain unbiased estimates of accuracy and bootstrap tests (5,000 samples) to determine which brain areas made reliable contributions to prediction.

##### Model Validation

To validate the models, we applied it to analogous “ground truth” and inference brain activity during the auditory-only and visual-only trails. These validation maps also had five levels of ‘ground truth’ emotion intensity and five levels of inferred emotion intensity. Validation trials were “held out,” meaning that they were not included in training or tested on during training.

There were two validation sets-one for each model–a “ground truth” validation set, and an inference validation set. A prediction-outcome correlation (partial *r*) between the model and each validation image was calculated for each subject (N = 100). Then we averaged participant’s partial *r* values and compared them within and across the validation sets. The validity of the model was established, first, by applying it to its own validation set (i.e., “ground truth” model was tested on the “ground truth” validation set) and, next, by testing if the average performance was positively greater than zero in a two-tailed single sample *t*-test.

To further validate the models, we tested its specificity to its own validation set. We did this by applying the model to the opposite validation set (i.e., “ground truth” model was tested on the inference validation set). Then we tested if the model’s average performance was higher on its own validation set versus the opposite validation set via a two-tailed paired *t*-test. We expected the “ground truth” model to have higher prediction-outcome correlation on the “ground truth” validation set than on the inference validation set. Likewise, we expected the inference model to have higher prediction-outcome correlation on the inference validation set than the “ground truth” validation set.

#### Characterization of the model pattern weights

The unthresholded predictive weight map (see Fig. 1) was input into the NeuroSynth Image Decoder (https://neurosynth.org/decode/) to quantitatively compare it to images in the Neurosynth database. This allows us to assess what behaviors and functions are most associated with our patterns of brain activity, across published literature. The top 50 terms that loaded onto the map were pruned down to remove single brain regions and redundant concepts (like theory of mind and ‘tom’). Then a word cloud was constructed of the top 20 terms. The networks and functions in the NeuroSynth database which were most similar to the model are depicted in the word cloud (Figs. 2 & 3), and the words are scaled by strength of similarity.

To verify that the “ground truth” and inference models are not reducible to general processing of affect, we compared our models’ patterns with previously published and publicly available models of affect and emotion. We used cosine similarity, a multivariate method of assessing the similarity between two vectors, to make these comparisons between our models’ pattern weights and eight published models of various components of PINES (Chang et al., 2015), NPS (Wager et al., 2013), the Vicarious Pain Signature (VPS; Krishnan et al., 2016), Empathic Care & Empathic Distress (Ashar et al., 2016), Social Rejection (Woo et al., 2014), Galvanic Skin Response (GSR; Eisenbarth, Chang, & Wager, 2016), and Heart Rate (Heart; Eisenbarth, Chang, & Wager, 2016; see Supplementary Fig. 5). These models are available freely online (https://github.com/canlab). Cosine similarity ranges from − 1 to 1, where values closer to 0 mean the two vectors are orthogonal or perpendicular to each other. When the value is closer to 1, it means the angle is smaller and images/brain patterns are more similar. When the value is closer to −1, it means the images are more opposite.

#### Comparison of the “ground truth” and inference maps

We tested the separability of the “ground truth” and inference subject-level intensity beta maps with a linear support vector machine (SVM; C = 1, optimizer = Adam (adaptive moment estimation), LOO-CV). This classification was performed across each level of intensity, and a receiver operating characteristic (ROC) curve was calculated across all the CV folds (within subject; Fig. 4). In this analysis, a classification accuracy significantly greater than chance indicates that the activity patterns are linearly separable, and therefore, represent unique neural processes (see Reddan, Wager, & Schiller, 2018). To better understand the brain regions that distinguish between “ground truth” and inference, the predictive voxel weights for the classifier trained at the highest level of intensity (level 5) was bootstrapped (5,000 samples) and thresholded (FDR *q* < 0.05) so that the brain regions where “ground truth” and inference maximally diverge could be compared (Fig. 4B). This thresholding was repeated also for the classifier trained at the lowest level of intensity (level 1) as a sanity check (Supplementary Fig. 7).

#### Model Alignment and Empathic Accuracy

To test how our models relate to an observer’s empathic accuracy, we took the dot-product between each model and individual subject’s (N = 100) maps of empathic accuracy. Then we correlated the resulting dot-products from low empathic accuracy trials and high empathic accuracy trials across all participants. Finally, the alignment analysis was validated on empathic accuracy maps from the visual-only and auditory-only trials, which are independent from the “ground truth” and inference model training data, to ensure that results were not biased. Two-tailed *z*-tests of the correlation difference were used to compare alignment scores because these scores are correlations.

During first-level modeling, the empathic accuracy regressors may overlap in time with the “ground truth” and inference regressors because empathic accuracy is partially derived from target and observer ratings. Therefore, first we confirmed the separability of the “ground truth” and inference subject-level beta maps from the empathic accuracy subject-level beta maps before testing how the alignment of the “ground truth” and inference models are related to empathic accuracy. The separability of the “ground truth” and inference beta maps from the empathic accuracy beta maps at two levels of empathic accuracy (low accuracy and high accuracy) were confirmed using a linear SVM (C = 1, optimizer = andre, LOO-CV). This classification was performed across each level of intensity vs the low and high empathic accuracy maps. The “ground truth” and inference maps were separable from the low and high empathic accuracy maps (accuracy range = 57% – 67%; SE = 1.5–1.6%; p < 0.0001).

#### COI

The all of the authors of this manuscript certify that they have no affiliations with or involvement in any organization or entity with any financial interest (such as honoraria; educational grants; participation in speakers’ bureaus; membership, employment, consultancies, stock ownership or other equity interest; and expert testimony or patent-licensing arrangements) or non-financial interest (such as personal or professional relationships, affiliations, knowledge or beliefs) in the subject matter or materials discussed in this manuscript.

## Results Summary

- The “ground truth” emotional state of another person can be predicted from an observer’s brain activity.
- An observer’s inferences about another person’s emotional state can also be predicted from the observer’s brain activity.
- Neural patterns predictive of “ground truth” and inference are completely dissociable.
- When our brain-based patterns of socioemotional “ground truth” and inference align, participants yield more accurate inferences.

## INCLUSION AND DIVERSITY

We worked to ensure gender balance in the recruitment of human subjects. We worked to ensure ethnic or other types of diversity in the recruitment of human subjects. We worked to ensure that the study questionnaires were prepared in an inclusive way. One or more of the authors of this paper self-identifies as an underrepresented ethnic minority in their field of research or within their geographical location. One or more of the authors of this paper self-identifies as a gender minority in their field of research.
